# Quality of acute internal medicine: A patient-centered approach. Validation and usage of the Patient Reported Measure-acute care in the Netherlands

**DOI:** 10.1371/journal.pone.0242603

**Published:** 2020-12-01

**Authors:** Marjolein N. T. Kremers, Elsemieke E. M. Mols, Yvonne A. E. Simons, Sander M. J. van Kuijk, Frits Holleman, Prabath W. B. Nanayakkara, Harm R. Haak

**Affiliations:** 1 Department of Health Services Research, and CAPHRI School for Public Health and Primary Care, Aging and Long Term Care, Maastricht, The Netherlands; 2 Department of Internal Medicine, Máxima MC, Veldhoven/Eindhoven, The Netherlands; 3 Department of KEMTA, Maastricht University Medical Center+, Maastricht, The Netherlands; 4 Department of Internal Medicine, Amsterdam UMC, Location Academic Medical Center, Amsterdam, The Netherlands; 5 Section General and Acute Internal Medicine, Amsterdam Public Health Research Institute, Department of Internal Medicine, Amsterdam UMC, Location VU University Medical Center, Amsterdam, The Netherlands; 6 Department of Internal Medicine, Division of General Internal Medicine, Maastricht University Medical Center+, Maastricht, The Netherlands; Radboud University Medical Center, NETHERLANDS

## Abstract

**Background:**

Providing high quality care is important and has gained more attention since the introduction of value-based healthcare. Value should be measured by outcomes achieved, relevant for patients. Patient-centeredness is one domain for quality improvement determined by the Institute of Medicine, aiming to deliver care responsive to the patient. The development and implementation of patient reported outcome- and experience measures can be used for this goal. Recently, we developed the Patient Reported Measure (PRM)-acute care, based on five relevant domains to evaluate and improve the quality of care in the Emergency Department (ED).

**Objective:**

To validate the PRM-acute care, in order to evaluate and improve patient-centered care in the ED.

**Methods:**

We performed a prospective questionnaire-based study. Patients ≥18 years presenting for internal medicine in the ED were eligible. The validity of the PRM-acute care was evaluated according to the COSMIN-criteria. We performed hypotheses testing to evaluate construct validity. The perceived quality of care was evaluated by statistical analysis.

**Results:**

Face- and content validity was evaluated based on previously performed research and deemed good. Construct validity was supported by demonstrated differences between subgroups; patients with severe symptoms had a higher perceived quality of care. The correlation between overall satisfaction and the total mean score of the PRM-acute care (r = 0,447, p = 0.01) was significant. Overall, patients reported a mean perceived quality of care of 4.67/6.0.

**Conclusion:**

The PRM-acute care is a valid instrument to measure the perceived quality of care in an acute setting for internal medicine patients. Additionally, patients reported a good perceived quality of care in the ED with scores ranging from moderate to well for each of the relevant domains. Therefore, we believe that the PRM-acute care can be implemented in daily practice to evaluate the perceived quality of care and to improve the quality of acute care.

## Introduction

The increasing attention that providing high quality care receives since the introduction of value-based healthcare creates a need for transparency of healthcare quality [1]. Value in health care should always be defined around the customer in a well-functioning care system, therefore value is measured by the outcomes achieved that are relevant for patients [2]. The Institute of Medicine determined patient-centeredness, defined as providing care that is respectful of and responsive to individual patient preferences, needs and values, and ensuring that patients`values guide all clinical decisions, to be one of six domains for measuring and improving healthcare quality [3].

Patient-centeredness is important for all healthcare domains, including the field of acute medicine. The emergency department (ED) is a busy environment characterised by rapid triage, acute conditions and a high turnover. As a consequence, an ED visit can be considered as a stressful life event and associated with adverse effects on the patient’s emotional state [4]. It is important that patients in the ED receive high-quality care and experience it as such. A way to assess the perceived quality of care by patients is the routine use of Patient-Reported Measures (PRMs), which consist of measures of satisfaction with outcomes of care and measures of experiences of care [5, 6]. So, Patient-Reported Outcomes (PROs) and patients perceptions of experiences whilst receiving care, known as Patient-Reported Experiences (PREs), are combined in PRMs. PROs and PREs are directly obtained from the patient without the interference of a clinician and pertain to the patients’ health, quality of life, and functional status associated with healthcare [7].

Many Patient-Reported Outcome Measures have been developed over the past years, being either generic, disease specific or patient specific. Their measurement properties often restrict successful use in different settings. Since the patient population in the ED is heterogeneous and often lacks a diagnosis, many disease and patient specific PROMs are not suitable for use in this setting [8]. In particular, patients presenting for internal medicine often suffer from multiple chronic conditions and present with non-specific complaints, which may explain why commonly used indicators do not reflect relevant outcomes for this specific group of patients [9]. In a previous study, we determined the PROs relevant to internal medicine patients in an acute setting. Five core domains were identified, namely relief of symptoms, understanding the diagnosis, understanding the treatment plan, reassurance and patient experiences [9]. Experiences were determined as a relevant domain, because in the patients’ perception of quality of care, both outcomes and experiences play an important role. While researchers and doctors try to distinguish Patient Reported Outcomes from Patient Reported Experiences, patients do not. So, based on these five domains, we developed a PRM-acute care in order to assess the perceived quality of acute care for internal medicine patients.

Acute care in the Netherlands is mainly provided by General Practitioners (GPs) in primary care and via EDs. To gain access to hospital care, including EDs, patients are required to have a referral from a GP or be directly transferred by an ambulance [10]. Due to increasing numbers of patients presenting in the ED with a higher age, comorbidity and therefore often a greater case complexity, the speciality acute medicine was recognised in 2012 as a subspecialty of internal medicine. The number of patients presenting for internal medicine increased slightly from 2013 to 2016. In 2013, on average 3,824 patients (range: 1,227–10,403) presented per hospital to the ED for internal medicine, compared to 4,343 patients (range: 1,418–29,426) in 2016 [11].

In this study, we primarily aim to assess the validity of the PRM-acute care in internal medicine patients and secondly, to gain insight into the current perceived quality of acute care, with the overarching goal to use the PRM-acute care in daily practice and improve patient-centered care in the ED.

## Methods

### Study design

We performed a prospective questionnaire-based study in two hospitals in the Netherlands (Máxima MC, Veldhoven and Amsterdam UMC, location VUmc), as part of the PROMESQUE trial. Within this trial, a baseline measurement and a consecutive intervention study was planned. This study concerns the baseline measurement. The study protocol was approved by the Medical Ethics Committees of the participating hospitals.

### Selection of participants

Patients were included between January 5 and March 12, 2020. Due to regulations during the COVID-19 crisis, the inclusion of patients was terminated in both hospitals. All patients ≥18 years presenting for internal medicine in the ED were eligible for inclusion. Patients were asked by their treating physician to participate in the study. Patients who were unable to participate due to a language barrier, inability to understand the questionnaire, or severity of illness, were excluded at the discretion of the treating physician. Patients willing to participate were approached by a researcher and written informed consent was obtained. The PRM-acute care was presented to participants during admission or at home by phone, 12 to 72 hours after arrival in the ED. This timeframe was selected with the planned intervention study in mind, because the presence of researchers in the ED and visible measurements might influence the daily practice in the ED (Hawthorne effect) and as a consequence the perceived quality of care.

### Development of the PRM-acute care

The development of the PRM-acute care was based on the previously determined five relevant domains for patients presenting for internal medicine in the ED, namely relief of symptoms, understanding the diagnosis, understanding the therapeutic plan, reassurance, and patient experiences [9]. A concept questionnaire was developed and presented in three focus groups to 16 different experts in the field of acute medicine by a trained interviewer (MK) to explore the face- and content validity. Thereafter, 15 patients were interviewed to identify face- and content validity of the questionnaire and subsequently a cognitive interview was performed by a researcher trained in qualitative interviewing (MK) in order to ensure that the questions were considered relevant and understood by patients, and to determine whether each question generated the information intended by the researchers. Patients were selected by purpose sampling. For all interviews a topic list was established and audio records were made. As a result, the PRM-acute care was finalised and consisted of 11 questions covering the five domains (Questionnaire in Dutch in S1 Appendix, Questionnaire in English in S2 Appendix). Answers to questions were scored on a Likert scale with a range of 1 to 6, except questions concerning symptoms, these were scored on a Numeric Rating Scale (NRS) with a range from 0 to 10.

The scoring model for the PRM-acute care was based on qualitative interviews with 10 patients assessing the importance of the five domains, which resulted in a total score consisting of the mean of all the reported domains. A maximum of one missing domain was accepted for analysing the questionnaire. The domain score was calculated as the mean score of the questions of that domain. One missing question per domain was accepted. Domain scores were defined as missing when the domain had more than one missing question, or if the domain score could not be calculated (in the case of domain ‘relief of symptoms’). Most domain scores were adopted from the results on the Likert-scales ranging 1–6. However, two exceptions were made. Firstly, within the domain ‘understanding the diagnosis’ a grade 0 was given when a patient reported not receiving any explanation about their diagnosis. Secondly, to establish the score of the domain ‘symptom relief’, we calculated the percentage difference between the severity of symptoms at arrival and discharge from the ED, and converted these into a 1–6 score, as is shown in the table in S1 Table. This grading system was based on an expert discussion and on literature, which defined the minimum clinically important difference in acute pain as 30% [12, 13].

### Measurements

#### Perceived quality of care

Participants were asked to recall their ED visit and complete the PRM-acute care. Hospitalised patients completed the PRM-acute care on paper, preferably by themselves. If necessary, caregivers or a researcher assisted without interfering in the interpretation of the patient. Patients at home received a link to the online PRM-acute care. Overall satisfaction was scored as a report mark, ranging from 1 to 10.

#### Visit and patient characteristics

Destination after ED visit, arrival and discharge date and time were extracted from the electronic patient file. Length of stay in the ED (LOS-ED) was calculated. Baseline characteristics, such as gender, age, living situation and educational level were obtained from all included patients. During the first month of the study, baseline characteristics such as gender, age and destination after ED, were also collected from patients who were not included in the study. All data were stored in a web-based database (Castor EDC).

### Data analysis

#### Validity testing

The PRM-acute care is based on a formative model, as perceived quality of care is determined by the five relevant domains [14, 15]. The validation of the PRM-acute care was executed in accordance with the COSMIN-criteria applying to a formative model [14, 16–18]. Firstly, face and content validity was evaluated based on our previous study and strengthened by additional cognitive interviews in this study, as mentioned in ‘development of the PRM-acute care’. Face validity is the extent to which a test is subjectively viewed as covering the concept it purports to measure. Content validity refers to the extent to which a measure represents all facets of a given construct.

Furthermore, hypotheses testing was used to assess construct validity. Construct validity refers to the degree to which a measure actually measures the theory it purports. Hypotheses were proposed with the objective to demonstrate differences in scores between subgroups, which would establish construct validity. Previous research indicates that differences related to scores regarding quality of care are present [19]. Amongst others, a positive relationship was found between patient satisfaction of care and higher age, lesser education, trust in the medical care centre and good communication between patients and healthcare professionals [19–21]. Moreover, a negative correlation between the experience of pain and patient satisfaction is demonstrated [21, 22]. However, due to mandatory guidelines on pain management [23], early pain recognition has gained much attention in the Netherlands which leads to prompt treatment and patient satisfaction. Lastly, a negative correlation between LOS-ED and patient satisfaction, has been reported [24, 25]. Considering these findings, we propose several hypotheses regarding differences in perceived quality of care between subgroups, namely: 1) Older patients perceive a higher quality of care, 2) Patients with lower education (middle-level applied education or lower) perceive a higher quality of care, 3) Patients arriving in the ED with severe complaints (graded as 8–10), will perceive higher quality of care than patients with mild complaints (graded as 0–4), 4) Patients with a LOS-ED ≥ 4 hours, perceive lower quality of care than patients with a LOS-ED <4 hours. In addition, we explored whether differences in perceived quality of care existed between hospitalised and patients discharged directly from the ED and between patients presenting during weekdays or weekends. Moreover, while the PRM-acute care is based on a formative model, we expect that differences in perceived quality in subgroups may only be present in specific domains. Therefore, statistical analysis of differences between subgroups regarding domain scores were executed.

Lastly, with the aim of strengthening the construct, we analysed whether the total score of the PRM-acute care and the individual domains correlates with the overall satisfaction of the ED-care, graded using a report mark (range 1–10).

### Statistical analysis

Patient and ED characteristics were analysed using descriptive statistics. Total- and domain scores were reported using the mean, standard deviation (SD) and 95% confidence interval (CI). To asses differences in the perceived quality of care between subgroups, unpaired T-tests and linear regression tests were used. To evaluate differences between subgroups on domain scores, the Mann-Whitney U test was used for the following domains: relief of symptoms, understanding the diagnosis, understanding the treatment plan and reassurance. An unpaired T-test was used to analyse the domain ‘experiences’. A Spearman’s rho was used to analyse the correlation between the overall satisfaction and relief of symptoms, understanding the diagnosis, understanding the treatment plan and reassurance. The correlation between the overall satisfaction and the domain ‘experiences’ was analysed using a Pearson’s rho. A p-value of 0.05 was considered significant. All analyses were performed using IBM SPSS version 26.0 for Windows.

## Results

### Patient characteristics

We included 81 patients, of which 47 were men (58%) as is shown in Table 1. The response rate, measured during the first three weeks of the study, was 86.6%. The mean age of the study sample was 68 years (range 26–93). All patients lived at home, 27 (33.3%) patients lived alone and 54 (66.7%) lived together. Of all patients, 57 patients (70.4%) were treated in MMC and 24 (29.6%) patients in A-UMC. Seventy patients (86.4%) were hospitalised after their ED visit and 11 patients (13.4%) were directly discharged from the ED. Seventy patients (86.4%) were seen during week-days, whereas 11 patients were seen during weekends (13.6%).

The study participants differed in gender and destination after ED visit compared to the patients who were not included. More men participated in the study (58% vs. 42%, p = 0.019) and more patients were hospitalised in the study group (86.4% vs. 13.6%, p <0.001). Patients were mostly not included because they were not asked by the treating physician to participate.

**Table 1:** Patient characteristics.

	Number of patients	Percentage (%)
Included Patients	81	
**Sex**		
*Male*	47	58.0
*Female*	34	42.0
**Age**		
*18–44 years*	8	9.9
*45–64 years*	22	27.2
*65–79 years*	34	42.0
*≥ 80 years*	17	21.0
**Living situation**		
*Living at home*, *single*	27	33.3
*Living at home*, *together*	54	66.7
*Nursing home*	0	0
**Level of education**		
*No education*	0	0
*Primary education*	8	9.9
*Middle-High school*	25	30.9
*Middle level applied education*	22	27.2
*Higher education*	24	29.6
Missing	2	2.5
**Institute visited**		
*MMC*	57	70.4
*VUMC*	24	29.6
**Time of presentation**		
*Weekdays*	70	86.4
*Weekends*	11	13.6
**Length of stay**		
*0>4 hours*	50	61.7
*≥ 4 hours*	31	38.3
**Destination**		
*Discharge*	11	13.6
*Admission*	70	86.4
**Initial graded severity of complaints**		
*0–4*	11	13.6
*5–7*	18	22.2
*8–10*	52	64.2

#### Face- and content validity

Face- and content validity was partly established in our previous study [9]. In this study, professionals in acute care recognised all domains and questions as relevant. No new themes came up during the cognitive interviews with patients and all questions were deemed relevant. Minor adjustments in the questionnaire were made based on these interviews. Additionally, we observed that all five domains were equally important to patients.

#### Construct validity

Patients experiencing severe symptoms had a higher mean total score as was shown by linear regression. The total mean score increased on average by 0.08 for each point increase in severity of symptoms (p = 0.006). The associations between age, gender, educational level, LOS-ED, discharge, and day of presentation with perceived quality were not statistically significant (Table 2). Subsequently, we evaluated differences between subgroups in each domain. Most differences were found among the subgroups in the domain ‘understanding the diagnosis’ as presented in Table 2. Patients who received less education had a greater perceived understanding of the diagnosis (mean 4.94, SD 1.39) than patients with a higher education (mean 4.14, SD 1.9) as shown by a Mann-Whitney U test (p = 0.01). Furthermore, patients with a LOS-ED < 4hours (mean 5.02, SD 1.41) scored on average higher in this domain than patients with a LOS-ED ≥ 4 hours (mean 4.18, SD 1.74, p = 0.003). Additionally, a linear regression model showed a significant association between the degree of understanding the diagnosis and the initial severity of symptoms (p = 0.005). Other significant differences were found in the domain ‘relief of symptoms’. Patients who experienced more severe symptoms on arrival in the ED, reported the biggest relief of symptoms. The domain score for relief of symptoms increases with 0.24 with each point increase on the NRS-scale, p = 0.001. Lastly, in the domain ‘patient experiences’ differences were found between admitted and discharged patients as admitted patients (mean 5.36, SD 0.53) reported higher scores than the discharged patients (mean 4.97, SD 0.45), p = 0.02.

**Table 2:** Results of hypotheses testing on total score and separate domains.

	Total Score			Relief of symptoms			Understanding the diagnosis			Understanding the treatment plan			Experiences			Reassurance		
**Higher age leads to higher perceived quality of care**	*R*^*2*^	*CI*	*P-value*	*R*^*2*^	*CI*	*P-value*	*R*^*2*^	*CI*	*P-value*	*R*^*2*^	*CI*	*P-value*	*R*^*2*^	*CI*	*P-value*	*R*^*2*^	*CI*	*P-value*
	0.00	(-0.01–0.01)	0.891	0.01	(-0.01–0.03)	0.307	0.00	(-0.03–0.02)	0.985	0.01	(-0.01–0.01)	0.946	0.00	(-0.01–0.01)	0.943	0.01	(0.02–0.01)	0.442
**Lesser education leads to higher perceived quality of care**	*Mean difference*	*CI*	*P-value*	*Effect size*	*Mean difference*	*P-value*	*Effect size*	*Mean difference*	*P-value*	*Effect size*	*Mean difference*	*P-value*	*Mean difference*	*CI*	*P-value*	*Effect size*	*Mean difference*	*p-value*
	0.26	(-0.05–0.58)	0.104	0.04	0.11	0.558	**0.73**	**-0.08**	**0.012**	0.05	-0.07	0.496	0.13	(-0.13–0.40)	0.339	0.24	0.53	0.142
**LOS >4h leads to a lower perceived quality of care**	*Mean difference*	*CI*	*P-value*	*Effect size*	*Mean difference*	*p-value*	*Effect size*	*Mean difference*	*P-value*	*Effect size*	*Mean difference*	*P-value*	*Mean difference*	*CI*	*P-value*	*Effect size*	*Mean difference*	*P-value*
	0.22	(-0.08–0.52)	0.15	0.12	-0.39	0.292	**1.00**	**0.84**	**0.003**	0.04	0.20	0.573	0.23	(-0.01–0.48)	0.057	0.00	0.23	0.867
**More complaints/pain leads to higher perceived quality of care**	*R*^*2*^	*CI*	*P-value*	*R*^*2*^	*CI*	*P-value*	*R*^*2*^	*CI*	*P-value*	*R*^*2*^	*CI*	*P-value*	*R*^*2*^	*CI*	*P-value*	*R*^*2*^	*CI*	*P-value*
	**0.09**	**(0.03–0.14)**	**0.006**	**0.14**	**(0.10–0.39)**	**0.001**	**0.10**	**(0.07–0.36)**	**0.005**	0.00	(-0.05–0.08)	0.666	0.01	(-0.3–0.08)	0.314	0.00	(-0.08–0.14)	0.594
**Difference between admission and discharge**	*Mean difference*	*CI*	*P-value*	*Effect size*	*Mean difference*	*P-value*	*Effect size*	*Mean difference*	*P-value*	*Effect size*	*Mean difference*	*P-value*	*Mean difference*	*CI*	*P-value*	*Effect size*	*Mean difference*	*P-value*
	0.31	(-0.11–0.72)	0.150	0.00	0.40	0.881	0.15	0.29	0.241	0.02	0.13	0.708	**0.40**	**(0.06–0.74)**	**0.023**	0.30	0.64	0.100
**Difference between weekdays and weekends**	*Mean difference*	*CI*	*P-value*	*Effect size*	*Mean difference*	*P-value*	*Effect size*	*Mean difference*	*P-value*	*Effect size*	*Mean difference*	*p-value*	*Mean difference*	*CI*	*P-value*	*Effect size*	*Mean difference*	*P-value*
	-0.09	(-0.52–0.33)	0.661	0.02	0.02	0.695	0.00	-0.35	0.975	0.16	0.06	0.237	-0.19	(-0.54–0.16)	0.274	0.00	0.12	0.853
**Difference between men and women**	*Mean difference*	*CI*	*P-value*	*Effect size*	*Mean difference*	*P-value*	*Effect size*	*Mean difference*	*P-value*	*Effect size*	*Mean difference*	*p-value*	*Mean difference*	*CI*	*P-value*	*Effect size*	*Mean difference*	*P-value*
	-0.22	(-0.54–0.04)	0.09	*0*.*29*	*-0*.*49*	*0*.*113*	***0*.*95***	***-0*.*4***	***0*.*004***	*0*.*02*	*-0*.*01*	*0*.*711*	*0*.*06*	*(-0*.*16–0*.*033)*	*0*.*479*	*0*.*02*	*-0*.*29*	*0*.*677*

**Fig 1:**
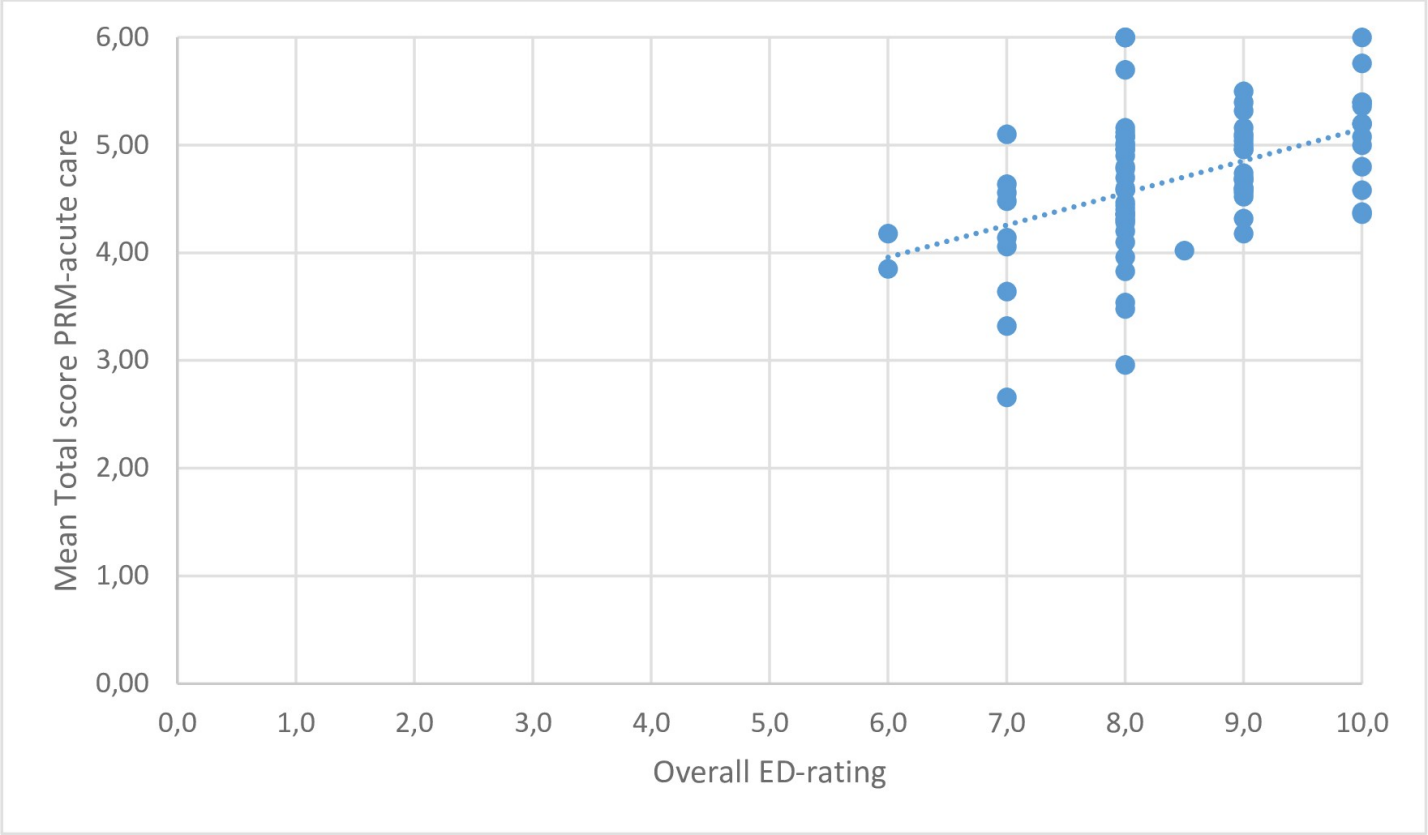
Correlation between total mean score and overall satisfaction in the ED.

In order to strengthen the construct, the correlation between the mean total score of the perceived quality of care and the overall satisfaction of the ED-care was tested. The mean total score was correlated to the graded overall satisfaction as is shown in Fig 1 (r = 0.447, p = 0.01). Additionally, the scores of all domains except the domain ‘relief of symptoms’ were correlated with the overall satisfaction of ED-care, as analysed using Spearman’s rho and Pearson’s rho as presented in Table 3.

**Table 3:** Correlation between overall satisfaction and individual domains.

Domain	Correlation co-efficient	N	P-value
Relief of symptoms	0.09	73	0.442
Understanding the diagnosis	0.32	78	0.004
Understanding the treatment plan	0.34	80	0.002
Experiences*	0.37	80	0.001
Reassurance	0.35	80	0.002

*All tests were Spearman’s rho, except for the domain ‘experiences’

#### Perceived quality

The total score of the PRM-acute care was calculated in all patients (n = 81). The mean total score for all patients was 4.67 (95% CI 4.53–4.82) with a range from 2.66 to 6.00 (Table 4). The overall satisfaction of ED-care was 8.4/10, (range 6 to 10). Evaluation of scores per domain showed a mean score in the domain ‘relief of symptoms’ of 3.03 (95% CI 2.68–3.35) as presented in Table 4. Seventy-four out of 81 patients responded to both questions within this domain. Two patients did not experience any symptoms during arrival and discharge, whereas five patients did not answer one of the two questions. These records were excluded. The domain ‘understanding the diagnosis’ was scored by 79 patients and had a mean score of 4.66 (95% CI 4.30–5.02). The domain ‘understanding the treatment plan was scored with a mean of 5.33 (95% CI 5.17–5.49) and answered by all patients. All patients reported on the domain ‘patient experiences’ and revealed a mean score of 5.31 (95% CI 5.19–5.43). Patients graded the domain ‘reassurance’ (n = 81) with a mean score of 4.93 (95% CI 4.67–5.18). A graphic overview of the distribution of scores within the domains is presented in the figure in S1 Fig.

**Table 4:** Mean scores per domain.

Domain	n =	Mean	SD	95% CI
Relief of symptoms	74	3.03	1.40	2.70–3.35
Understanding the diagnosis	79	4.66	1.61	4.30–5.02
Understanding treatment plan	81	5.33	0.72	5.17–5.49
Experiences	81	5.31	0.54	5.19–5.43
Reassurance	81	4.93	1.16	4.67–5.18
**Total score**	**81**	**4.67**	**0.653**	**4.53–4.82**

## Discussion

This study is the first use of PRMs in the ED in the Netherlands, consisting of both outcome and experience measures. We examined the validity of the PRM-acute care following the COSMIN-criteria for a formative construct-model. Intensive previous research formed the basis of the PRM-acute care [9], including semi-structured interviews with both healthcare professionals and patients, which was followed by cognitive testing in this study. Therefore, we deem the face- and content validity as good. Additionally, we conclude that the construct validity is adequate. This is supported by the demonstrated differences in perceived quality of care between subgroups and the correlation between the overall satisfaction of the ED-care and total score of the PRM-acute care.

The most notable difference in perceived quality of care exists between patients experiencing severe symptoms on arrival int the ED and patients with less severe symptoms. The severity of symptoms appears to be positively correlated with the total mean score of the PRM-acute care. Boudreaux et al also showed that the satisfaction level of ED-care was higher in those with serious illnesses or emergency needs [26]. We believe that our findings can be explained by the increased attention of healthcare professionals for patients who are obviously suffering and the perception of a more favourable throughput time in these patients.

Furthermore, we found differences between subgroups in specific domains of the PRM-acute care, which is important as in a formative model all domains determine the perceived quality of care. Firstly, patients who received less education did perceive a better understanding of the diagnosis. These results are in line with previous findings, showing that patients with less education tend to have a higher perceived quality of care and patient satisfaction [27–29]. Secondly, patients with a LOS-ED <4 hours had a better understanding of the diagnosis. This could be due to the complexity of the situation of patients with a LOS-ED ≥4 hours and the number of consultants involved. Research shows that the complexity of the case and the number of consultants involved are correlated with the LOS-ED [30, 31]. Lastly, admitted patients were more satisfied with their ED experiences than discharged patients. This seems to be caused by a lower satisfaction with the waiting time in discharged patients. An association between perceived waiting time vs expected waiting time on patient satisfaction has been indicated previously [32].

Moreover, we found a positive correlation between the overall satisfaction with the ED-care and the total mean score of the PRM-acute care. A positive correlation between the overall score of the ED and the domains ‘understanding of the diagnosis’, ‘understanding of the treatment plan’ and ‘experiences’ was also found. These correlations show that an increase in understanding the diagnosis or treatment plan, as well as better experiences, may induce an increase in overall ED-rating, which is in accordance with previous research [28, 33]. The domain ‘relief of symptoms’ did not significantly correlate with overall satisfaction, which may be due to the selected scoring method, which was based on literature regarding only pain instead of heterogeneous symptoms. Moreover, the found correlations endorse the underlying formative model as the rated perceived quality of care increases even if only one of the domains shows an increase. However, as the correlation between overall satisfaction and the total score of the PRM-acute care knows a wide distribution, grading overall satisfaction by a report mark cannot fathom the complexity of perceived quality of care. Therefore, a more elaborate model is needed, such as the PRM-acute care model.

Due to the study design and construct model we were not able to evaluate the reliability and thus were limited to the evaluation of the face-, content- and construct validity. The validating measurements are less well-known for a formative model and therefore might seem limited. However, this does not imply that the methods we used to validate the PRM-acute care are less reliable or validating [18].

Following a demonstrated validity of the PRM-acute care, we evaluated the perceived quality of ED-care for internal medicine patients. Overall, the perceived quality of care in the EDs was good, with a mean score of 4.67/6.0. As the Dutch healthcare system is known as outstanding in Europe, with the Netherlands being the only country consistently among the top 3 of the European Health Consumer Index [34], these results may be an example of the high quality of care in the Netherlands. Performing this study internationally would be of interest in order to evaluate the association between the perceived quality of care and the ranking in the European Health Consumer Index.

Within the specific domains, the most remarkable findings concern the domain ‘understanding the treatment plan’. In our study, patients perceive their understanding of the treatment plan good to very well. However, many studies have shown that patients regularly do not understand their treatment plan or discharge instructions [35, 36]. More importantly, most patients appear to be unaware of their lack of understanding, which might be also the case in our study and an explanation for the high scores [37, 38]. So, based on our results and the literature, it is important for physicians in the ED to be aware of the possible dissimilarity between perceived understanding and real understanding. The teach-back method could be used as a tool to confirm understanding and improve recall, especially in discharged patients [39].

Evaluating the use of the PRM-acute care, we believe implementing this questionnaire into daily practice is feasible. Our study did not reveal major problems during the inclusion process, besides the challenge of reaching discharged patients. Almost all of the included patients filled out all questions and did not report any difficulties. The questionnaire is short, consisting of only 11 questions, which is not time consuming (around 10 minutes). Another study in the Netherlands also showed the feasibility of using a PROM in an acute medical unit. Patients especially appreciated the fact that their view was taken into account [40].

### Limitations

On account of the spread of COVID-19, patient based research was suspended indefinitely. Subsequently, the smaller sample size could have contributed to the inability to demonstrate differences between subgroups. One might also suggest differences are simply not there, because the healthcare system in the Netherlands is known to be outstanding for several years [34, 41]. This could also be the cause for high scores of perceived quality of care among various subgroups. Especially the distribution between hospitalised and discharged patients is not optimal to identify differences between these groups, even though this distribution represents daily practice. We experienced that physicians were prone to forget to approach patients in the ED, when not reminded by a researcher. Since the admitted patients could be approached on a later moment in time, this has led to a skewed distribution between discharged and admitted patients and has contributed to selection bias. Additionally, it is possible that during busy periods in the ED physicians were more prone to forget to include patients. This may have led to a greater perceived quality of care amongst the included patients, as busy periods may affect the length of stay and communication negatively.

Secondly, patients were asked to fill in the questionnaire within 12–72 hours after their ED visit, because of an intended future intervention study. This delay can affect the memory of the patient and cause recall bias [42]. To limit recall bias, patients should preferably fill out the questionnaire immediately after their ED visit.

Lastly, patients’ opinions about the concept questionnaire were inventoried during individual interviews. Organising focus groups aiming to select relevant questions could have resulted in a more profound discussion of this topic.

## Conclusion

The PRM-acute care is a valid instrument to measure the perceived quality of healthcare in an acute setting for internal medicine patients. Additionally, patients reported a good perceived quality of care in the ED and a score ranging from moderate to well was given for each of the relevant domains.

### Recommendations

We recommend the use of the PRM-acute care in the ED to evaluate the perceived quality of care for internal medicine patients in order to improve the quality of care. As the PRM-acute care is able to indicate within which domain(s) improvements are needed, tailor-made adjustments can be directly implemented for every single patient and in the ED as a whole. Cross-cultural validation should be executed to validate this instrument for use in international settings. When it is not possible to execute the PRM-acute care, the use of an overall satisfaction score of the ED-care can be considered as a screening tool for the perceived quality of care. We only recommend this for severely time constrained situations, as patients who perceived a low quality of care can be missed and it will remain unclear in which domain improvements could be beneficial.

## Supporting information

S1 Appendix Questionnaire in Dutch. (DOCX)

S2 Appendix Questionnaire in English. (DOCX)

S1 Table Scoring domain relief of symptoms. (DOCX)

S1 Fig Overview of distributions of scores per domain. (DOCX)

S1 Dataset Dataset PRM-acute care. (XLSX)
