# Supplementary material for: Quality of acute internal medicine: A patient-centered approach. Validation and usage of the Patient Reported Measure-acute care in the Netherlands
Source: PLoS One. 2020 Dec 1;15(12):e0242603. doi: 10.1371/journal.pone.0242603 (PMC7707480; doi:10.1371/journal.pone.0242603)
Supplement: S1 Appendix — (DOCX) [file pone.0242603.s001.docx]

**VRAGENLIJST: DE ERVAREN KWALITEIT VAN DE SPOED EISENDE HULP**

U bent behandeld op de Spoed Eisende Hulp voor het specialisme interne geneeskunde. Wij willen graag weten hoe u de zorg ervaren heeft en of u goed geholpen bent op de Spoed Eisende Hulp.

Wilt u terugdenken aan uw bezoek aan de Spoed Eisende Hulp en onderstaande vragen beantwoorden door het meest passende cijfer te omcirkelen?

|  |  | **Geen last Zeer veel last** |
| --- | --- | --- |
| 1. Hoeveel last had u van uw klachten bij binnenkomst op de Spoed Eisende Hulp? |  | 0 1 2 3 4 5 6 7 8 9 10 |
| 1. Hoeveel last had u van uw klachten bij het verlaten van de Spoed Eisende Hulp? |  | 0 1 2 3 4 5 6 7 8 9 10 |
|  |  |  |

|  |  | **Helemaal niet** | **Vrijwel niet** | **Matig** | **Behoorlijk** | **Goed** | **Volledig** |
| --- | --- | --- | --- | --- | --- | --- | --- |
| 1. Begreep u de uitleg op de Spoed Eisende Hulp over de oorzaak van uw klachten? |  | 1 | 2 | 3 | 4 | 5 | 6 |
|  |  | *Ik heb geen uitleg over de oorzaak van mijn klachten gekregen* | | | | | |
| 1. Wist de arts wat de oorzaak van uw klachten was op de Spoed Eisende Hulp? |  | 1 | 2 | 3 | 4 | 5 | 6 |
|  |  |  |  |  |  |  |  |
| 1. Begrijpt u waarom de onderzoeken en/of behandelingen op de Spoed Eisende Hulp uitgevoerd zijn? (bijv. bloed prikken, infuus) |  | 1 | 2 | 3 | 4 | 5 | 6 |
| 1. Begrijpt u wat er nog voor u (of uw klachten) gedaan moet worden tijdens opname in het ziekenhuis of thuis? |  | **Helemaal niet**  1 | **Vrijwel niet**  2 | **Matig**  3 | **Behoorlijk**  4 | **Goed**  5 | **Volledig**  6 |
| 1. Voelde u zich gerustgesteld na uw bezoek aan de Spoed Eisende Hulp? |  | 1 | 2 | 3 | 4 | 5 | 6 |
|  |  |  |  |  |  |  |  |
| 1. Bent u tevreden over de totale duur van uw verblijf op de Spoed Eisende Hulp? |  | 1 | 2 | 3 | 4 | 5 | 6 |
| 1. Voelde u zich veilig tijdens uw verblijf op de Spoed Eisende Hulp? |  | 1 | 2 | 3 | 4 | 5 | 6 |
| 1. Werd er door de zorgverleners naar u geluisterd tijdens uw verblijf op de Spoed Eisende Hulp? |  | 1 | 2 | 3 | 4 | 5 | 6 |
| 1. Had u vertrouwen in de deskundigheid van de zorgverleners op de Spoed Eisende Hulp? |  | 1 | 2 | 3 | 4 | 5 | 6 |
|  |  | **Zeer slecht Zeer goed** | | | | | |
| 1. Welk cijfer zou u de Spoed Eisende Hulp geven op een schaal van 0-10? |  | 0 1 2 3 4 5 6 7 8 9 10 | | | | | |

Om verschillen tussen patiëntengroepen te kunnen meten, willen we nog enkele persoonlijke kenmerken van u weten. Wilt u hiervoor onderstaande vragen beantwoorden?

1. Wat is uw leeftijd? 4. Wat is uw hoogst genoten opleiding?

_______________ Geen opleiding

Lagere school / basisschool

1. Wat is uw geslacht? Lager beroepsonderwijs / MAVO/ VMBO

- Man HAVO / VWO
- Vrouw Middelbaar beroepsonderwijs (MBO)

Hoger beroepsonderwijs (HBO)

1. Wat is uw woonsituatie? Wetenschappelijk onderwijs (WO)

- Thuiswonend
  - Alleen
  - Samenwonend
- Verblijf in verzorgingshuis
- Verblijf in verpleeghuis
