# Supplementary material for: Quality of acute internal medicine: A patient-centered approach. Validation and usage of the Patient Reported Measure-acute care in the Netherlands
Source: PLoS One. 2020 Dec 1;15(12):e0242603. doi: 10.1371/journal.pone.0242603 (PMC7707480; doi:10.1371/journal.pone.0242603)
Supplement: S2 Appendix — (DOCX) [file pone.0242603.s002.docx]

## **Questionnaire: the perceived quality of care in the Emergengy Department**

You are treated in the Emergency Department for the specialty of internal medicine. We would like to know how you perceived the delivered care and if you feel you are treated well in our Emergency Department.

Could you please recall your Emergency Department visit and answer the following questions?

|  | | |  | **No complaints Very severe complaints** | | | | | |
| --- | --- | --- | --- | --- | --- | --- | --- | --- | --- |
| 1. What was the severity of your complaints on arrival at the Emergency Department? | | |  | 0 1 2 3 4 5 6 7 8 9 10 | | | | | |
| 1. What was the severity of your complaints on departure from the Emergency Department? | | |  | 0 1 2 3 4 5 6 7 8 9 10 | | | | | |
|  |  | **Not at all** | | | **Barely** | **Moderate** | **Fairly** | **Good** | **Completely** |
| 1. Did you understand the explanation in the Emergency Department about the cause of your complaints? |  | 1 | | | 2 | 3 | 4 | 5 | 6 |
|  |  | *I did not get an explanation about the cause of my complaints* | | | | | | | |
|  |  |  | | |  |  |  |  |  |
| 1. Do you understand why additional diagnostics and treatments were executed in the Emergency Department? |  | 1 | | | 2 | 3 | 4 | 5 | 6 |
| 1. Do you understand the next steps in the treatment of your condition, during admission or at home? |  | 1 | | | 2 | 3 | 4 | 5 | 6 |
| 1. Did you feel reassured after your visit of the Emergency Department? |  | 1 | | | 2 | 3 | 4 | 5 | 6 |
|  |  |  | | |  |  |  |  |  |
| 1. Are you satisfied with the total length of stay in the Emergency Department? |  | **Not at all**  1 | | | **Barely**  2 | **Moderate**  3 | **Fairly**  4 | **Good**  5 | **Completely**  6 |
| 1. Did you feel safe during your visit in the Emergency Department? |  | 1 | | | 2 | 3 | 4 | 5 | 6 |
| 1. Did the healthcare professionals listen attentively to you, during your stay in the Emergency Department? |  | 1 | | | 2 | 3 | 4 | 5 | 6 |
| 1. Did you have trust in the expertise of the healthcare professionals in the Emergency Department?   ***Additional questions*** |  | 1 | | | 2 | 3 | 4 | 5 | 6 |
|  |  | **Very poor Very good** | | | | | | | |
| 1. How would you grade the Emergency Department in general? (on a scale form zero tot ten) |  | 0 1 2 3 4 5 6 7 8 9 10 | | | | | | | |

***Patient characteristics***

1. Gender
2. Age
3. Living situation
4. Educational level
