# Supplementary material for: Quality of acute internal medicine: A patient-centered approach. Validation and usage of the Patient Reported Measure-acute care in the Netherlands
Source: PLoS One. 2020 Dec 1;15(12):e0242603. doi: 10.1371/journal.pone.0242603 (PMC7707480; doi:10.1371/journal.pone.0242603)
Supplement: S1 Table — (DOCX) [file pone.0242603.s003.docx]

*Table: scoring of the domain ‘relief of symptoms’*

| Scoring domain 1: Symptom relief | Difference (%) |
| --- | --- |
| 1 | < 0% |
| 2 | 0% |
| 3 | 0-30% |
| 4 | 30-50% |
| 5 | 50-80% |
| 6 | > 80% |
