# Supplementary material for: Quality of acute internal medicine: A patient-centered approach. Validation and usage of the Patient Reported Measure-acute care in the Netherlands
Source: PLoS One. 2020 Dec 1;15(12):e0242603. doi: 10.1371/journal.pone.0242603 (PMC7707480; doi:10.1371/journal.pone.0242603)
Supplement: S1 Fig — (DOCX) [file pone.0242603.s004.docx]

## Figure: graphic overview of the distribution of scores per domain
